# Secretome analysis revealed adaptive and non-adaptive responses of the *Staphylococcus carnosus femB* mutant

**DOI:** 10.1002/pmic.201400343

**Published:** 2015-01-21

**Authors:** Mulugeta Nega, Linda Dube, Melanie Kull, Anne-Kathrin Ziebandt, Patrick Ebner, Dirk Albrecht, Bernhard Krismer, Ralf Rosenstein, Michael Hecker, Friedrich Götz

**Affiliations:** 1 Microbial Genetics, Interfaculty Institute of Microbiology and Infection Medicine, University of TübingenTübingen, Germany; 2 Institute for Microbiology, University of GreifswaldGreifswald, Germany; 3 Cellular and Molecular Microbiology Division, Medical Microbiology and Hygiene Institute, University of TübingenTübingen, Germany

**Keywords:** Cytosolic proteins, FemB, Microbiology, Peptidoglycan, Secretome, *Staphylococcus carnosus*

## Abstract

FemABX peptidyl transferases are involved in non-ribosomal pentaglycine interpeptide bridge biosynthesis. Here, we characterized the phenotype of a *Staphylococcus carnosus femB* deletion mutant, which was affected in growth and showed pleiotropic effects such as enhanced methicillin sensitivity, lysostaphin resistance, cell clustering, and decreased peptidoglycan cross-linking. However, comparative secretome analysis revealed a most striking difference in the massive secretion or release of proteins into the culture supernatant in the *femB* mutant than the wild type. The secreted proteins can be categorized into typical cytosolic proteins and various murein hydrolases. As the transcription of the murein hydrolase genes was up-regulated in the mutant, they most likely represent an adaption response to the life threatening mutation. Even though the transcription of the cytosolic protein genes was unaltered, their high abundance in the supernatant of the mutant is most likely due to membrane leakage triggered by the weakened murein sacculus and enhanced autolysins.

## 1 Introduction

In many organisms where the classical secretion systems are not involved in the excretion of cytosolic proteins, the “non-classical protein secretion” takes place. Proteins undergoing this secretion show no simple sequence motifs except that they are more disordered in structure than those remaining in the cytoplasm [1]. In *Staphylococcus aureus*, over 20 typical cytosolic proteins are excreted, starting already in the exponential phase and it appears to be more pronounced in clinical isolates than in the non-pathogenic staphylococcal species [2,3]. Various *S. aureus* mutants have been analyzed with respect to the release of cytoplasmic proteins. For example, in the *atl* (major autolysin) mutant, cytosolic proteins were hardly found in the supernatant, but were entrapped within the huge cell clusters of this mutant [2]. In the wall teichoic acid deficient *tagO* mutant, with its increased autolysis activity, excretion of cytosolic proteins was increased compared to the wt, confirming the importance of autolysis in excreting cytosolic proteins.

Here, we investigated a *femB* deletion mutant of *Staphylococcus carnosus*, which has a shortened glycine interpeptide bridge in the murein structure. The factors essential for the expression of methicillin resistance (fem), encode the FemABX peptidyl transferases involved in non-ribosomal pentaglycine interpeptide bridge biosynthesis [4,5]. While femX is essential, *Tn* mutants, but no deletion mutants, could be generated in *S. aureus*. Both mutants showed a reduced peptidoglycan (PGN) cross linking and glycine content, decreased lysostaphin susceptibility, reduced whole-cell autolysis, increased sensitivity to β-lactam antibiotics [6], an aberrant placement of cross walls, and stunted cell separation [7] showing key functions of the pentaglycine interpeptide bridge.

We could construct a *femB* deletion mutant in *S. carnosus* in which the *femAB* operon is orthologous to that of *S. aureus* [8]. The most striking phenotype of the femB mutant was the massive release of proteins into the culture supernatant. The excreted proteins could be classified into two groups: those secreted via the canonical Sec pathway, and those representing typical cytosolic proteins lacking a signal sequence. The results suggest that the release of cytosolic proteins is due to the altered PGN structure, which makes the cell envelope leaky enough for the release of cytosolic proteins.

## 2 Materials and methods

### 2.1 Bacterial strains, growth conditions, and oligonucleotide primers

*S. carnosus* strains TM300 [9,10], its deletion mutant Δ*femB::ermB*, the complementary mutant Δ*femB::ermB* (pPSHG5femB), *Escherichia coli* strain DH5α [11], and *Micrococcus luteus* DSM 20030^T^ [12,13] were cultivated at 37°C and shaken in basic medium (BM; 1% soy peptone, 0.5% yeast extract, 0.5% NaCl, 0.1% glucose, and 0.1% K_2_HPO_4_; pH 7.4). When appropriate, BM was supplemented with 10 μg/mL chloramphenicol, 2.5 μg/mL erythromycin, or 100 μg/mL ampicillin. Oligonucleotide primers used for cloning and Northern blot analysis are listed in Supporting Information Table 1.

### 2.2 Preparation of extracellular proteins for preparative 2DE

**Figure 1:**
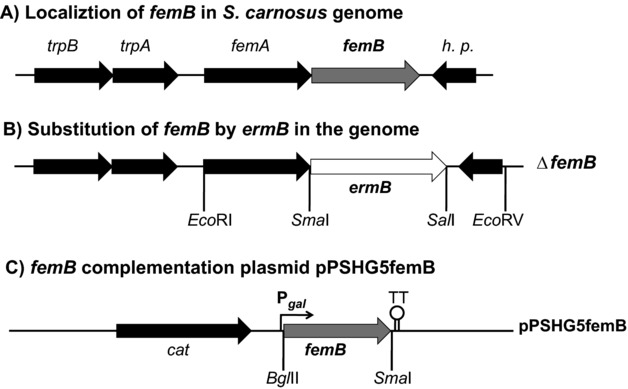
Construction of the *femB* deletion mutant and complementation. (A) Location of the *fem* operon in the chromosome of *S. carnosus* and (B) the *femB* deletion mutant (Δ*femB*::*ermB*). (C) Complementation plasmid pPSHG5femB. *cat*: chloramphenicol resistance gene; *ermB*: erythromycin resistance gene; *trpA*, *trpB*: tryptophan synthetase alpha and beta chain; *h.p*.: hypothetical protein.

Cells were harvested at a comparable time point of the growth phase (Fig.1 and Supporting Information Table 2) by centrifugation at 9000 × *g* for 15 min at 4°C. Extracellular proteins in the culture supernatant were precipitated with 10% trichloroacetic acid overnight at 4°C, subsequently pelleted by centrifugation at 9000 × *g* for 40 min at 4°C, and washed eight times with ethanol. The protein pellet was dried and resuspended in an appropriate volume of rehydration buffer consisting of 8 M urea, 2 M thiourea, 4% w/v CHAPS, 1% DTT, and 0.7% pharmalyte, pH 3–10 and centrifuged at 16 000 × *g* for 15 min at room temperature. The protein concentration was determined using Bradford assay according to the manufacturer's instructions (Bio-Rad Laboratories, München, Germany). For 2D PAGE, 500 μg of secreted proteins was applied.

### 2.3 Preparation of cytosolic proteins for preparative 2DE

Cells of 40 mL cultures were harvested at two time points (Fig.1 and Supporting Information Table 2) and centrifuged for 15 min at 9000 × *g* at 4°C. The pellets were then resuspended in 1 mL ice-cold TE buffer (10 mM Tris and 1 mM EDTA; pH 7.5) and disrupted by homogenization using glass beads and TissueLyser (Qiagen) twice for 30 s at 30 Hz. To remove cell fragments and insoluble proteins, the cleared lysate was centrifuged for 20 min at 20 000 × *g* at 4°C. The protein concentration was determined using Bradford assay. For 2D PAGE, 500 μg was used and mixed with rehydration buffer to a final concentration containing 8 M urea, 2 M thiourea, 4% w/v CHAPS, 1% DTT, and 0.7% pharmalyte (pH 3–10).

### 2.4 2D-PAGE and computational analysis

Protein patterns of the *S. carnosus* wt and the *femB* mutant were compared visually and quantitatively with Delta2D software (DECODON) after 2D PAGE was performed as described in earlier studies [14,15]. For each condition, three independent experiments were performed. Only statistically reproducible differences were included in the results. For identified proteins, several analyses were performed. The theoretical localization of proteins was predicted with PSORT (http://www.psort.org/psortb/), the prediction of N-terminal signal sequences was performed with SignalP version 3.0 (http://www.cbs.dtu.dk/services/SignalP/), and the non-classical secretion of proteins was predicted with SecretomP version 2.0 (http://www.cbs.dtu.dk/services/SecretomeP/). The theoretical molecular weight (MW) and p*I* for mature proteins without any signal sequence were calculated using the p*I*/MW tool (http://www.expasy.org/tools/pi_tool.html).

### 2.5 Supporting information

Construction of the *S. carnosus femB* deletion mutant, construction of *femB* expression plasmid, antimicrobial susceptibility testing, purification and analysis of PGN, analysis of proteins in the supernatant, analysis of membrane and cytosolic fractions by SDS-PAGE, Western blot analysis, fluorescence microscopy, RNA isolation and northern blot analysis, and protein identification by MS are described as Supporting Information.

## 3 Results

### 3.1 Construction and characterization of the *femB* deletion mutant in *S. carnosus* TM300

**Figure 2:**
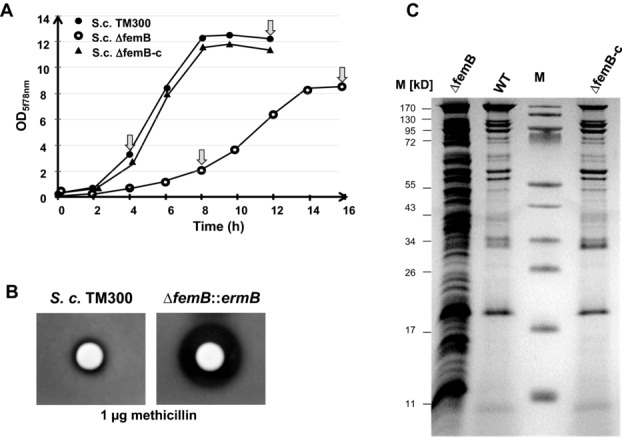
Comparative phenotypic features of *S. carnosus*, its *femB* mutant, and the complementary mutant. (A) Growth was severely affected in the *femB* mutant; arrow indicates sampling time for analysis. (B) Agar diffusion assay showing *femB* mutant methicillin susceptibility. (C) SDS-PAGE of culture supernatant proteins; cells were cultivated for 13 h in the presence of 0.25% galactose. wt: wild type; S.c. TM300; ΔfemB: femB deletion mutant; ΔfemB-c: mutant complemented with *pPSHG5femB;* M: marker proteins.

FemB (Sca_1020) of *S. carnosus* TM300 revealed 82% identity and 92% similarity to FemB (SAOUHSC_01374) of *S. aureus* NCTC 8325 and the *femAB* genes are organized in an operon downstream of the tryptophan synthase genes (*trpBA*) (Fig.1A). In *S. carnosus* TM300, the deletion mutant was created by replacing *femB* with an erythromycin cassette (*ermB*) [16]. The *femB* mutant was complemented with the plasmid pPSHG5femB, which was constructed by cloning *femB* under the control of a galactose-inducible promoter (Fig.1B). Growth deficiency of the *femB* mutant could be complemented by pPSHG5femB (Fig.1C and Fig.2A). It also showed an increased susceptibility to methicillin (Fig.2B) but high resistance to lysostaphin, with a more than 3000-fold increase in the MIC values from 0.01 to 32 μg/mL. This is in agreement with lysostaphin's cleavage preference for the pentaglycine interpeptide bridge [17].

**Figure 3:**
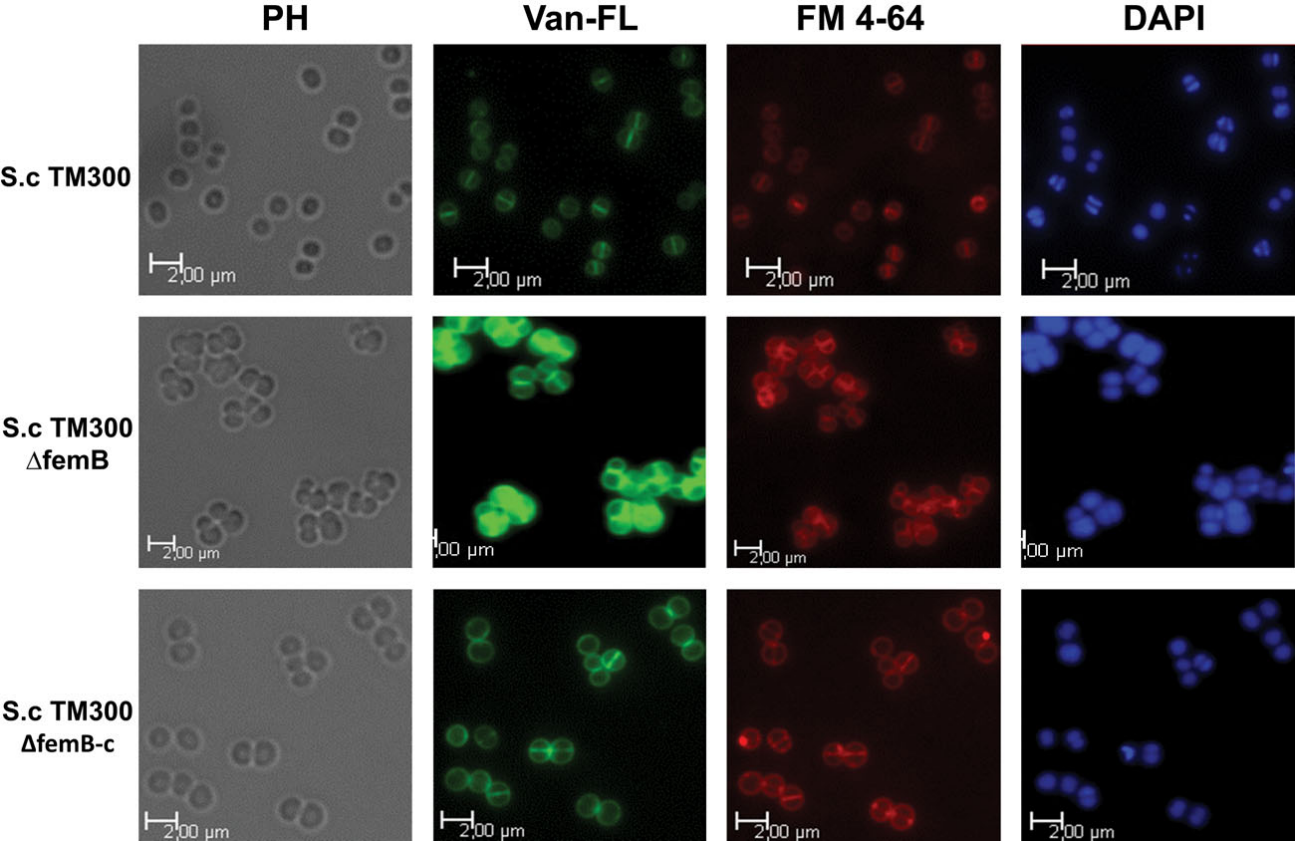
Microscopic analysis of *S. carnosus* and its *femB* mutant. *S.c*. TM300Δ*femB* forms large cell clusters. Cell wall staining with vancomycin shows intensive fluorescence particularly in the septum and membrane staining (FM 4–64) revealed intensified florescence. The DAPI stained nucleoid shows significant enlargement, whereas the wt (upper row) and the complementary mutant (lower row) looked roughly similar.

**Figure 4:**
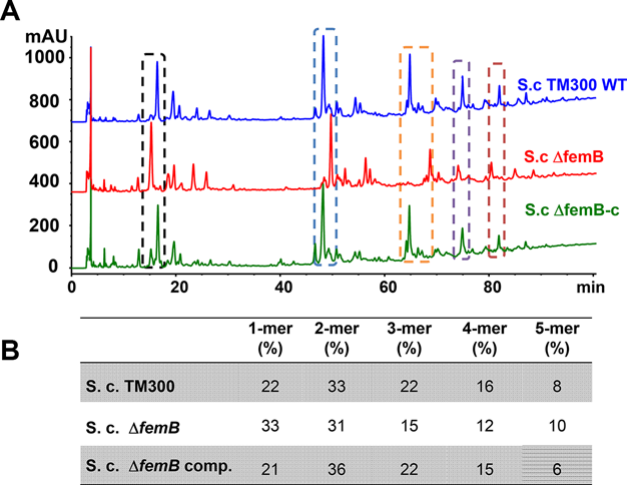
Peptidoglycan composition is altered in the *S. carnosus*Δ*femB* strain. (A) HPLC analysis of mutanolysin-digested PGN of the wild-type strain of *S. carnosus* TM300, the mutant *S.c*Δ*femB*, and the complemented mutant. (B) Eluted UV-absorbing peaks were integrated, and the corresponding muropeptides highlighted by the dotted area in (A) were quantified as a percentage of the total area of identified peaks. Dotted areas represent monomers to pentamers (left to right).

Phase and fluorescence microscopic analyses showed that cells of the *femB* mutant are clustered (Fig.3) suggesting a defect in daughter cell separation. Vancomycin staining (Van-FL) revealed a massive accumulation of fluorescence intensity in the *femB* mutant in the septum region suggesting that the degree of cross-linking of the PGN network was considerably decreased. As with vancomycin, fluorescence intensity with FM 4–64, a cell impermeant membrane stain, was increased in the septum region of the *femB* mutant, indicating increased penetration of the dye through the cell wall to the membrane site (Fig.3). Ultimately, DNA-staining with DAPI revealed enlarged DNA areas (nucleoid) in the mutant, which could have resulted from decreased chromosomal condensation (Fig.3). In all microscopic images, the *femB* mutant cells were enlarged, the average cell diameter was 132% increased compared to the wt; while the complementary mutant ΔfemB (PSHG5femB) closely resembles the wt (Fig.3). Comparative HPLC analysis of PGN isolated from the wt, Δ*femB*, and the complementary mutant showed that the mutant contained roughly 50% more of monomeric fragments than the wt, and less tri- and tetramer fragments (Fig.4), which indicates a decreased level of PGN crosslinking.

### 3.2 The *femB* mutant is characterized by the high abundance of secreted proteins

Another eye-catching phenotype of the *femB* mutant was the drastic increase of secreted proteins (Fig.2C). Quantitative analysis of these proteins in the exponential and stationary growth phases revealed that the protein content in the *femB* mutant was always roughly 5 to 6 times higher than in the wt, while the cytosolic protein content remained more or less the same (Table1 and Supporting Information Fig. 1). Comparative secretome analysis was performed to determine protein abundance in the supernatant of the *femB* mutant. Due to the growth rate difference (Fig.2A), protein samples for 2D-PAGE were taken at the exponential growth phase after 4 h (wt) and 8 h (*femB* mutant), as well as after 12 h (wt) and 16 h (*femB* mutant) for the stationary growth phase (Fig.2A and Supporting Information Fig. 2). Protein spots of each of three 2D gels of the wt and the *femB* mutant were analyzed by mass spectroscopy and 82 different proteins could be identified and quantified (Supporting Information Table 2).

**Table 1:** Protein content in supernatant and cytosol of wt and *femB* mutant

Strains	Harvest- time	OD_578_a)	Protein amount (μg/mL cell culture)	
			Supernatant a)	Cytosol a)
Exponential growth phase				
*S. c*. WT	4 h	4.5	0.87	22.60
*S. c*. Δ*femB*	8 h	3.3	5.74	22.28
Stationary growth phase				
*S. c*. WT	12 h	12.1	2.20	29.40
*S. c*. Δ*femB*	16 h	9.23	10.31	25.48

a) Indicates the mean values of the three measurements.

**Figure 5:**
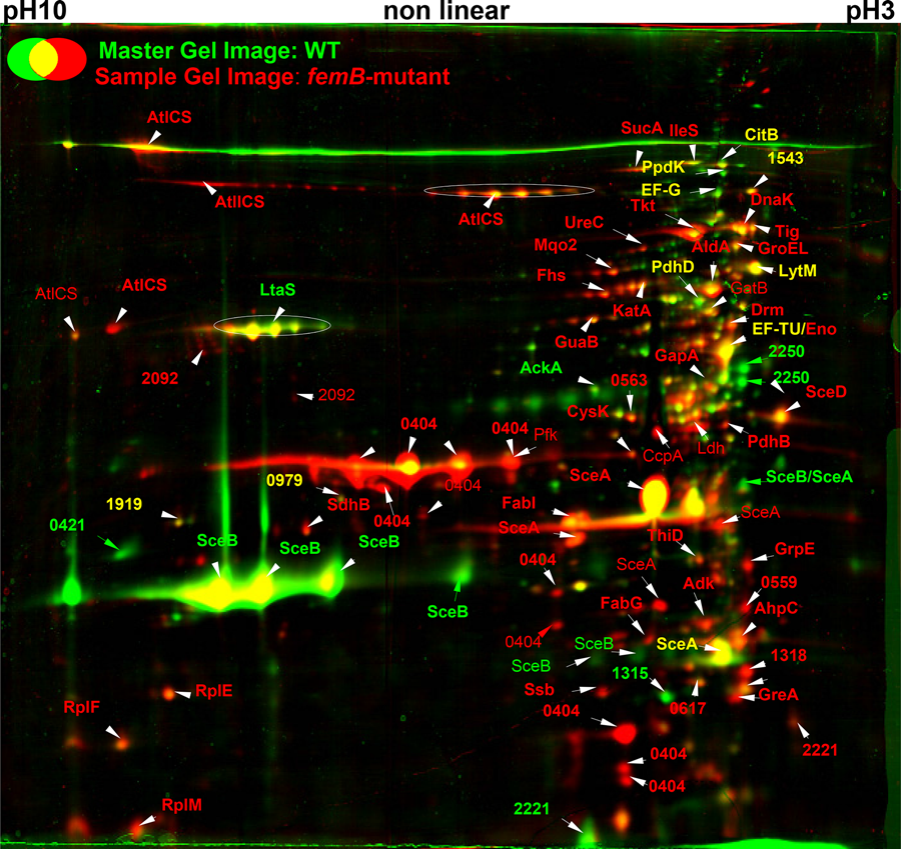
Differential 2D-PAGE of secretomes of *S. carnosus* wt and its *femB* mutant after 12 and 16 h incubation, respectively. Proteins (500 μg) of the culture supernatant were separated by 2D-PAGE and stained with colloidal coomassie silver blue and compared using the Delta2D image analysis software. The secretome of the WT (green) and *femB* mutant (red) were overlaid using Delta2D software. Dominant spots retain their colors and equally distributed ones showed yellow color. Protein spots were identified by MALDI-TOF MS.

Though the overall protein pattern was similar, the *femB* mutant showed a much higher protein quantity in most of the spots. Protein amounts were therefore compared using the Delta2D software (DECODON) for better visualization (Fig.5). Proteins in the wt were designated green and in the *femB* mutant red. Equal amount of proteins would then result in yellow spots. Thirty six selected protein spots showing the most significant differences in intensity between the wt and *femB* mutant were identified and characterized (Tables2 and 3).

**Table 2:** Proteins less abundant in the secretome of the *femB* mutant

Proteina)b)	Function	*S*. *carnosus* TM300 gene ID	*S. aureus* N315 homolog gene ID	*S. aureus* COL homolog gene ID	Ratio *femB* mutant/wild typec)	Signal sequenced)	MW (kDa)f)	p*I*f)
**Extracellular proteins**								
SceB^1^	SceB precursor	Sca_1790	SA2093 *ssaA*	SACOL2291	−8.0	+	25.3	7.9
SceB^2^	SceB precursor	Sca_1790	SA2093 *ssaA*	SACOL2291	−5.3	+	25.3	7.9
SceB^3^	SceB precursor	Sca_1790	SA2093 *ssaA*	SACOL2291	−2.8	+	25.3	7.9
Sca0421	Hypothetical protein	Sca_0421	−	−	−6.2	+	34.5	9.7
Sca2250	Hypothetical protein	Sca_2250	−	−	−9.2	+	37.2	4.5
**Membrane localization**								
LtaS^1^	Polyglycerol phosphate synthase LtaS	Sca_0366	SA0674	SACOL0778	−4.2	−	74.3	9.0
LtaS^2^	Polyglycerol phosphate synthase LtaS	Sca_0366	SA0674	SACOL0778	−3.5	−	74.3	9.0
LtaS^3^	Polyglycerol phosphate synthase LtaS	Sca_0366	SA0674	SACOL0778	−2.6	−	74.3	9.0
**Cytosolic proteins**								
AckA	Acetate kinase homolog	Sca_1316	SA1533	−	−5.8	−	43.6	5.5
Sca1315	Hypothetical protein	Sca_1315	SA1532	SACOL1759	−2.2	−	18.8	5.2

a) Subcellular localization was predicted using PSORTb.

b) Several identified spots of one protein were numbered.

c) Volume ratios in the range of 1 to ∞ indicate an increase of the volume of the respective protein spot and volume ratios in the range –1 to –∞ indicate a decrease of the volume of the respective protein spot. Only volume ratios ≥ 2 and ≤ −2 were defined as significant changes between the different strains.

d) Typical signal sequence was predicted using SignalP.

e) Non-classical secretion was predicted using SecretomeP.

f) Theoretical molecular weight (MW) and p*I* were calculated for mature proteins without signal sequence using MW/p*I* tools.

Superscripts 1–3 refer to differently processed protein spots.

**Table 3:** Proteins more abundant in the secretome of the *S. carnosus femB* mutant

Proteina)b)	Function	S. *carnosus* TM300 gene ID	*S. aureus* N315 homolog gene ID	*S. aureus* COL homolog gene ID	Ratio *femB* mutant/wild typec)	Signal sequenced)	Non-classical secretione)	MW (kDa)f)	p*I*f)
**Extracellular proteins**									
AtlCS^2^	Major autolysin precursor	Sca_0659	SA0905 *atlA*	SACOL1062	9.8	+	−	133.1	9.2
AtlCS^3^	Major autolysin precursor	Sca_0659	SA0905 *atlA*	SACOL1062	8.8	+	−	133.1	9.2
AtlCS^4^	Major autolysin precursor	Sca_0659	SA0905 *atlA*	SACOL1062	8.8	+	−	133.1	9.2
AtlCS^5^	Major autolysin precursor	Sca_0659	SA0905 *atlA*	SACOL1062	8.6	+	−	133.1	9.2
AtlCS^6^	Major autolysin precursor	Sca_0659	SA0905 *atlA*	SACOL1062	6.8	+	−	133.1	9.2
AtlCS^7^	Major autolysin precursor	Sca_0659	SA0905 *atlA*	SACOL1062	6	+	−	133.1	9.2
AtlCS^10^	Major autolysin precursor	Sca_0659	SA0905 *atlA*	SACOL1062	2.2	+	−	133.1	9.2
AtlCS^11^	Major autolysin precursor	Sca_0659	SA0905 *atlA*	SACOL1062	2.1	+	−	133.1	9.2
SceA	SceA precursor	Sca_1598	SA1898 *sceD*	SACOL2088	3.6	+	−	22.2	5.2
			SA2356 *isaA*	SACOL22584					
SceA^1^	SceA precursor	Sca_1598	SA1898 *sceD*	SACOL2088	3.3	+	−	22.2	5.2
			SA2356 *isaA*	SACOL22584					
SceA^2^	SceA precursor	Sca_1598	SA1898 *sceD*	SACOL2088	3.4	+	−	22.2	5.2
			SA2356 *isaA*	SACOL22584					
Sca0404	LysM family protein	Sca_0404	−	−	6.8	+	−	32.4	6.1
Sca0404^1^	LysM family protein	Sca_0404	−	−	17.8	+	−	32.4	6.1
Sca0404^2^	LysM family protein	Sca_0404	−	−	12.3	+	−	32.4	6.1
Sca0404^3^	LysM family protein	Sca_0404	−	−	4.1	+	−	32.4	6.1
Sca0404^4^	LysM family protein	Sca_0404	−	−	6.9	+	−	32.4	6.1
Sca0404^5^	LysM family protein	Sca_0404	−	−	6.1	+	−	32.4	6.1
Sca0404^6^	LysM family protein	Sca_0404	−	−	4.1	+	−	32.4	6.1
Sca0404^7^	LysM family protein	Sca_0404	−	−	4.9	+	−	32.4	6.1
Sca0404^8^	LysM family protein	Sca_0404	−	−	6.5	+	−	32.4	6.1
Sca0404^9^	LysM family protein	Sca_0404	−	−	4.5	+	−	32.4	6.1
Sca0404^10^	LysM family protein	Sca_0404	−	−	3.3	+	−	32.4	6.1
Sca2221	Hypothetical protein	Sca_2221	−	−	2.8	+	−	17.1	5.1
**Cell wall anchored proteins**									
Sca2092	Hypothetical protein	Sca_2092	−	−	2.7	+	−	48.1	7.9
Sca2092	Hypothetical protein	Sca_2092	−	−	13.66	+	−	48.1	7.9
**Membrane proteins**									
Fhs	Formate-tetrahydrofolate ligase homolog	Sca_1337	SA1553 *fhs*	SACOL1782	3.6	−	−	59.9	5.4
Fhs^1^	Formate-tetrahydrofolate ligase homolog	Sca_1337	SA1553 *fhs*	SACOL1782	5	−	−	59.9	5.4
GlpD	Aerobic glycerol-3-phosphate dehydrogenase homolog	Sca_0950	SA1142 *glpD*	SACOL1321	4.2	−	+	62.7	5.8
Lqo2	Putative lactate:quinone oxidoreductase	Sca_2266	SA2400 *mqo2*	SACOL2623	3.0	−	−	55.5	5.4
Lqo2^1^	Putative lactate:quinone oxidoreductase	Sca_2266	SA2400 *mqo2*	SACOL2623	4.0	−	−	55.5	5.4
SdhB	Succinate dehydrogenase iron-sulfur protein subunit homolog	Sca_0767	SA0996 *sdhB*	SACOL1160	6.3	−	−	30.8	6.5
**Cytosolic proteins**									
FabG	3-oxoacyl-(acyl-carrier protein) reductase	Sca_0854	SA1074 *fabG*	SACOL1245	3.1	−	−	26.1	5.2
FabI	Putative trans-2-enoyl-ACP reductase	Sca_0612	SA0869 *fabI*	SACOL1016	7.0		−	28.0	5.5
GapA	Glyceraldehyde-3-phosphate dehydrogenase	Sca_0424	SA0727 *gap*	SACOL1016	3.8	−	+	36.3	4.7
GrpE	Putative GrpE protein (HSP-70 cofactor)	Sca_1203	Sa1410 *grpE*	SACOL1638	2.3	−	+	23.0	4.3
GuaB	Putative inositol-monophosphate dehydrogenase	Sca_0049	SA0375 *guaB*	SACOL0460	2.2	−	−	52.8	5.5
KatA	Catalase	Sca_2336	SA1170 *katA*	SACOL0866	2.1	−	−	57.2	5.3
KatA^1^	Catalase	Sca_2336	SA1170 *katA*	SACOL0866	2.9	−	−	57.2	5.3
PdhB	Pyruvate dehydrogenase E1 component beta subunit homolog	Sca_0720	SA0944 *pdhB*	SACOL1103	10	−	−	35.2	4.6
RplC	50S ribosomal protein L3 homolog	Sca_1735	SA2047 *rplC*	SACOL_2239	2.2	−	+	23.7	9.6
RplE	50S ribosomal protein P5 homolog	Sca_1723	SA2035 *rplE*	SACOL2227	5.1	−	−	20.2	9.0
RplF	Probable 50S ribosomal protein L6	Sca_1720	SA2033 *rplF*	SACOL2224	3.5	−	+	19.6	9.5
RplJ	50S ribosomal protein L10 homolog	Sca_0195	SA0497 *rplJ*	SACOL0585	2.4	−	−	17.8	5.1
OdhA	Putatative 2-oxoglutarate dehydrogenase E1 component	Sca_1058	SA1245 *kgd*	SACOL1449	2.7	−	+	105.6	5.3
Tig	Trigger factor homolog	Sca_1281	SA1499 *tig*	SACOL1722	2.2	−	−	49.5	4.3
ThiD	Putative phosphomethylpyrimidine kinase	Sca_1595	SA1896 *thiD*	SACOL2085	2.1	−	−	29.8	5.3
Tkt	Putative transketolase	Sca_0983	SA1177 *tkt*	SACOL1377	3	−	−	72.8	5.0
TpiA	Triosephosphate isomerase homolog	Sca_0426	SA0729 *tpi*	SACOL0840	4.7	−	−	27.5	4.8
UreC	UreC urease alpha subunit homolog	Sca_1782	SA2084 *ureC*	SACOL2282	3.9	−	−	62.4	5.3
Sca0081	Putative intracellular protease/amidase	Sca_0081	−	−	6.8	−	−	25.7	6.7
Sca0559	Putative peptidyl-prolyl cis-trans isomerase	Sca_0559	SA0815	SACOL0957	2.1	−	−	21.8	4.4
Sca0563	NADH-dependent flavin oxidoreductase	Sca_0563	SA0817	SACOL0392	2.2	−	−	42.3	5.3
Sca1991	Pyruvate oxidase	Sca_1991	SA2327	SACOL0893	3.8	−	−	63.6	6.1

a) Subcellular localization was predicted using PSORTb.

b) Several identified spots of one protein were numbered.

c) Volume ratios in the range of 1 to ∞ indicate an increase of the volume of the respective protein spot and volume ratios in the range –1 to –∞ indicate a decrease of the volume of the respective protein spot. Only volume ratios ≥ 2 and ≤ −2 were defined as significant changes between the different strains.

d) Typical signal sequence was predicted using SignalP.

e) Non-classical secretion was predicted using SecretomeP.

f) Theoretical molecular weight (MW) and p*I* were calculated for mature proteins without signal sequence using MW/p*I* tools.

Superscripts refer to differently processed protein spots.

### 3.3 Few proteins show increased abundance in the wt secretome compared to the *femB* mutant

Only seven proteins were less abundant in the mutant secretome (Fig.5). Four of them belonged to signal peptide-dependent transported proteins: SceB, Sca0421, Sca2221, and Sca2250 (Table2). SceB showed 60% identity with Staphylococcal secretory antigen A (SsaA) of *Staphylococcus epidermidis* and *S. aureus*. SsaA is described in *S. epidermidis* as a highly antigenic protein [18]. The proteins Sca2250 and Sca0421 revealed no significant similarity to known proteins. Interestingly, the polyglycerol phosphate synthase LtaS (lipoteichoic acid synthase), a lipoteichoic acid biosynthesis enzyme, was also less abundant in the mutant. LtaS and its homologs in other Gram-positive bacteria were predicted to be polytopic membrane proteins with a large enzymatic domain located on the extracellular side of the bacterial membrane. According to this topological prediction, a cleaved fragment of the LtaS protein containing the complete enzymatic sulfatase domain was detected in the supernatant and cell wall-associated fractions in *S. aureus* [3,19]. Recently, the structure of the extracellular LtaS protein was determined and found to contain the complete enzymatic sulfatase [20]. Two typical cytosolic proteins, the acetate kinase homolog (AckA) and a hypothetical protein (Sca1315), were also found to be less abundant in the mutant secretome than in the wt (Table2).

### 3.4 Proteins showing increased abundance in the secretome of the *femB* mutant belong to three categories

#### 3.4.1 Murein hydrolases

From the 30 proteins analyzed that are more abundant in the *femB* mutant (Table3), four proteins belonged to signal peptide-dependent transported proteins: AtlCS, the major autolysin; SceA, which resembled SceD and IsaA of *S. aureus*; Sca0404, a LysM family protein; and Sca2221, a hypothetical protein. All of these enzymes are involved in murein turnover and daughter cell separation, and their increased production in the *femB* mutant is most likely a compensatory response to partially resolve the cell-wall interlinked cell clusters. AtlCS, the major autolysin, organized similarly to its homologs in *S. aureus* (AtlA) and *S. epidermidis* (AtlE) [21–23], is produced as a bifunctional precursor protein and functions primarily to hydrolyze the murein in the septum of the daughter cells catalyzing cell separation. The occurrence of multiple protein spots (Fig.5 and Table3) is most likely due to their processing in defined substructures of the precursor protein [22]. SceA belongs to the early and highly expressed exoproteins in *S. carnosus* [24], and its similarity to SceD (32%) and immunodominant antigen IsaA (37%) of *S. aureus* and *S. epidermidis* suggests that it is a cell wall hydrolase. IsaA and SceD are two putative lytic transglycosylases of *S. aureus* with autolytic activity. The inactivation of *sceD* resulted in impaired cell separation, as indicated by cell clumping [25]. Sca0404 belongs to the LysM family of proteins and is similar in size to autolysins Aae and Aaa [26,27] that contain repetitive sequences in their N-terminal portion that represent the PGN-binding domain (LysM) and a C-terminally located cysteine- and histidine-dependent amidohydrolase/peptidase (CHAP) domain with bacteriolytic activity in many proteins [28]. Lastly, Sca2221 is a small (17 kDa) protein, which shows no conspicuous similarity.

#### 3.4.2 Cytosolic proteins

The vast majority of increased protein spots in the *femB* mutant were typical cytosolic proteins (Table3). We identified 20 highly salient proteins, some of which represent typical enzymes of central metabolic pathways, such as FabG, FabI, GuaB, PdhB, OdhA, ThiD, Tkt, TpiA, UreC, Sca0081 (putative intracellular protease/amidase), Sca0563 (NADH-dependent flavin oxidoreductase), and Sca1991 (pyruvate oxidase). Others are involved in protein folding and oxidative stress situations—GrpE, Tig, Sca0559 (putative peptidyl-prolyl cis-trans isomerase), and KatA, while still others represent 50S ribosomal proteins such as RplC, RplE, RplF, and RplJ.

#### 3.4.3 Membrane-associated enzymes

Four membrane-associated enzymes increased in the culture supernatant: formate-tetrahydrofolate ligase (Fhs), Lqo, GlpD, and SdhB (Table3). Fhs transfers formyl groups to 10-formyl-tetrahydrofolate (formyl-THF). In anaerobic conditions, PFL (pyruvate formate lyase) is important as a formate donor [29]. GlpD (aerobic glycerol-3-phosphate dehydrogenase) is membrane-associated in *S. aureus* and strongly activated by detergents. In the *femB* mutant Lqo (earlier named Mqo2) was enhanced; it is required for the reassimilation of l-lactate during NO·-stress. Lqo is also critical to respiratory growth in l-lactate as a sole carbon source [30]. Finally, SdhB is part of the succinate dehydrogenase complex (Sdh) consisting of three subunits: a membrane-bound cytochrome b-558 (SdhC), a flavoprotein containing an FAD-binding site (SdhA), and an iron-sulfur protein with a binding region signature of the 4Fe-4S-type (SdhB) [31]. Sdh is part of the TCA cycle, which plays a central role in oxidative growth, and catalyzes the oxidation of succinate to fumarate by donating FADH_2_ for oxidative phosphorylation.

#### 3.4.4 Localization analysis of four typical cytoplasmic proteins

**Figure 6:**
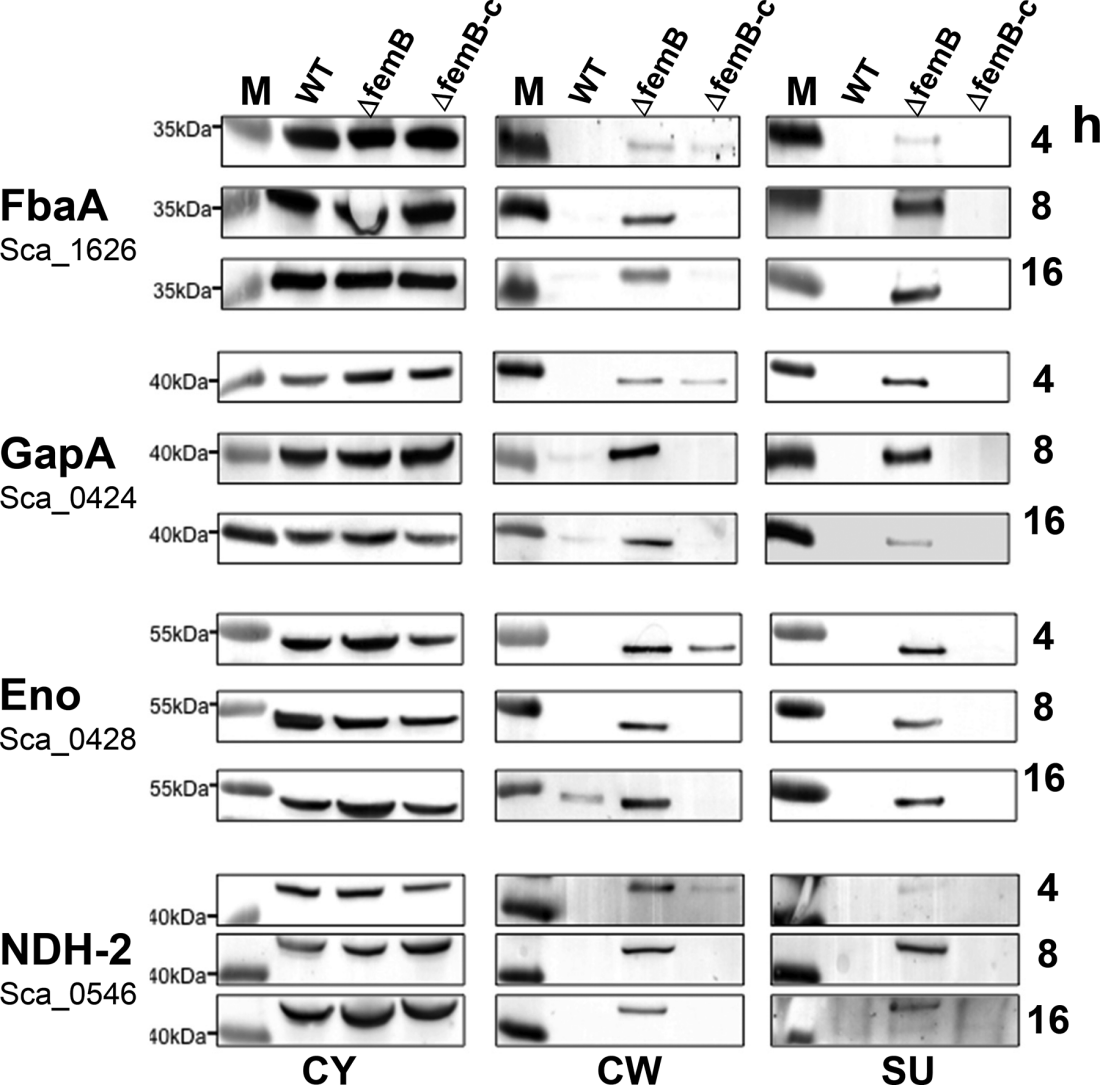
Localization of cytoplasmic marker proteins of *S. carnosus* wt, its *femB* mutant and the complementary mutant (femB-c) in the cytosole, cell wall, and supernatant by Western blot after 4, 8, and 16 h cultivation. The proteins fructose-bisphosphate aldolase (FabA), glyceraldehyde-3-phosphate dehydrogenase (GapA), enolase (Eno), and NADH-dehydrogenase (NDH-2) were each detected using specific antibodies. M: pre-stained molecular weight marker.

To verify the data of secretome analysis, we purified four typical *S. aureus*- specific cytosolic proteins, raised rabbit antibodies, and determined the presence of the target proteins in the cytosolic fraction, the cell wall fraction, and the culture supernatant by Western blotting (Fig.6). The antibodies cross-reacted with the highly conserved *S. carnosus* counterparts. The cytosolic proteins investigated were glyceraldehyde-3-phosphate dehydrogenase (GapA), enolase (Eno), fructose-1,6-bisphosphat-aldolase (FbaA), and NADH dehydrogenase (NDH-2). In the cytosolic fraction (CY), there was no marked difference in protein amounts between the wt, the *femB* mutant, and the complemented *femB* mutant. There was, however, a clear difference in the cell wall fraction (CW) and culture supernatant (SU) showing a significant increase in the *femB* mutant confirming that the cytosolic target proteins were abundantly exported.

#### 3.4.5 Transcription of genes encoding cell-wall lytic enzymes was up-regulated in the *femB* mutant, while that of cytosolic target genes was unchanged

Northern blot analysis of a selected set of genes encoding secreted and cytosolic proteins was performed to examine a possible correlation with the increased protein secretion. RNA was isolated from the wt, its *femB* mutant, and the complemented mutant in the exponential growth phase. The transcripts for the *sceA*, *sceB*, *sceD*, *atlCS*, and *sca*_0404 genes are clearly increased in the *femB* mutant, which correlates well with the increased amount of protein in the supernatant of the *femB* mutant (Supporting Information Table 3). Only SceB was an exception, as it was more abundant in the wt secretome. However, the transcription level of genes encoding the cytosolic proteins AhpC, GapA, KatA, Eno, and Tkt was essentially unchanged in the wt and the mutant, though these proteins were more abundant in the secretome of the mutant (Supporting Information Table 3).

## 4 Discussion

Here, we showed that the alteration of the cell wall structure in the *femB* mutant of *S. carnosus* had an enormous impact on morphology and physiology: expanded cells, retarded growth, high susceptibility to cell wall antibiotics, decrease of PGN crosslinking or unusually high secretion, and release of proteins into the culture supernatant. These pleiotropic effects suggest that the shortening of the interpeptide bridge from five to three glycine residues poses a life-threatening problem for the cells. The comparative analysis of secreted proteins in wt and *femB* mutant revealed that some proteins were less but the vast majority was more abundant in the mutant. The question is which of the differently expressed proteins in the secretome of the mutant is a response to adaptation or represents collateral damage.

It is very likely that the various Sec-dependent enzymes overexpressed in the *femB* mutant represent an adaptation response as the transcription of the corresponding genes is increased in the mutant. AtlCS, SceA, and Sca0404 represent murein hydrolyses. AtlCS belongs to the major staphylococcal autolysins Atl [21]. In staphylococci, Atl is crucial for daughter cell separation [32]. The other two secondary murein hydrolases, which were over-represented in the secretome of the *femB* mutant, were SceA and Sca0404. SceA is homologous to SceD and IsaA which represent two putative lytic transglycosylases in *S. aureus* [25] and Sca0404 belongs to the LysM family of proteins that represents a PGN-binding domain [33]. Because of the four- to nine-fold overexpression of the murein hydrolases AtlCS, SceA, and Sca0404 we assume that they represent a compensation reaction to accommodate the altered PGN structure in order to partially allow cell wall growth and daughter cell separation.

Proteins with a decreased prevalence in the secretome of the *femB* mutant were SceB, LtaS, AckA, and Sca1315. Particularly SceB and LtaS are interesting as their decreased production might also be part of the survival strategy of the *femB* mutant. SceB belongs to the prominently secreted exoproteins in *S. carnosus*; it is homologous to the *S. epidermidis* SsaA [18], which contains a cysteine- and histidine-dependent amidohydrolases and peptidases (CHAP) domain, which functions in some proteins as a l-muramoyl-l-alanine amidase or a d-alanyl-glycyl endopeptidase within the PGN [34,35]. Particularly, the d-alanyl-glycyl endopeptidase activity could be fatal in the *femB* mutant, as it would further decrease the degree of PGN cross-linking thus aggravating the already weakened murein network. Surprisingly, the membrane-associated LtaS was also decreased in the secretome of the *femB* mutant. Lipoteichoic acid (LTA), an important cell wall component of Gram-positive bacteria, is membrane-anchored via its lipid moiety. LtaS is required for LTA backbone synthesis and it has been shown recently that the enzyme accumulates at the cell division site [36]. Therefore, its presence in the secretome of the wt was not surprising, but its decreased presence in the secretome of the *femB* mutant was. In principle, we expected an increased LtaS expression in the *femB* mutant, as LTA synthesis is required for bacterial growth and cell division [37] and a decreased LtaS might worsen the growth defect of the *femB* mutant. Recently, it has been shown that LTA serves as a receptor for the Atl-repeats at the cross wall [38]. If the LTA content were decreased in the *femB* mutant, then back-binding of AtlCS to the cross wall would be affected; therefore the upregulation of AtlCS as a compensation reaction would make sense.

However, the vast majority of proteins overrepresented in the secretome of the *femB* mutant represent typical cytosolic proteins (Table3). With a few examples—FbaA, GapA, Eno, and Ndh-2—we showed in Western blots that these proteins are highly increased in the cell-wall fraction and the supernatant of the *femB* mutant (Fig.6). Interestingly, their quantity in the cytosolic fraction was essentially similar to that of the wt and the complemented mutant, which suggests that the level of gene expression should not differ much in the wt and mutant. Indeed, transcription analysis revealed no significant difference in the tested *ahpC*, *gapA*, *katA*, *eno*, and *tkt* genes (Supporting Information Table 3). Release of typical cytosolic proteins into the culture supernatant, also referred to as “non-classical protein excretion,” has been observed in many Gram-positive and Gram-negative bacteria such as staphylococci, streptococci, *Bacillus subtilis, Listeria monocytogenes*, or *E. coli*. In particular, glycolytic enzymes, chaperones, translation factors, or enzymes involved in detoxification of ROS were found in the supernatants by secretome analysis [39–44].

As there is no indication for an increased gene expression for the corresponding proteins, we don't think that they contribute much to the survival strategy of the *femB* mutant. We assume that the increased release of these proteins can be ascribed to a collateral damage of the altered cell wall structure and the increased autolysis activity.

## Abbreviations

BM: basic medium
CHAP: cysteine- and histidine-dependent amidohydrolase/peptidase
CY: cytosolic fraction
LTA: lipoteichoic acid
LtaS: lipoteichoic acid synthase
MW: molecular weight
PGN: peptidoglycan
PFL: pyruvate formate lyase
wt: wild type
