# Supplementary material for: Secretome analysis revealed adaptive and non-adaptive responses of the Staphylococcus carnosus femB mutant
Source: Proteomics. 2015 Jan 21;15(7):1268–79. doi: 10.1002/pmic.201400343 (PMC4409834; doi:10.1002/pmic.201400343)
Supplement: Supplementary file 1 [file pmic0015-1268-sd1.docx]

**Secretome analysis revealed adaptive and non-adaptive responses of the *Staphylococcus carnosus* *femB* mutant**

Mulugeta Nega^1#^, Linda Dube^1#^, Melanie Kull^1^, Anne-Kathrin Ziebandt^1^, Patrick Ebner^1^, Dirk Albrecht^2^, Bernhard Krismer^3^, Ralf Rosenstein^1^, Michael Hecker^2^, and Friedrich Götz^1*^

**Supporting Information**

**Material and Methods**

**Construction of the *S. carnosus femB* deletion mutant**

The erythromycin resistance gene *ermB* of Tn*551* was cut and purified from plasmid pEC3 [1] by SmaI/SalI digestion and ligated into the SmaI/SalI-digested temperature-sensitive shuttle vector pBT2 [1], which created plasmid pBTermB. Used primers are listed in Supporting Information Table 1. Primers 1koFemBEcoRIup and 2koFemBSmaIdo were used to amplify the upstream flanking region of *femB* (Sca_1020) from the chromosomal DNA of *S. carnosus* TM300. The polymerase chain reaction (PCR) product was cloned into the EcoRI/SmaI sites of pBT2ermB, which yielded pBT2ermBupfl. Primers 3koFemBSalIup and 4koFemBEcoRVdo were used to amplify the downstream flanking region of *femB*. The PCR product was cloned into the SalI/EcoRI sites of pBT2ermBupfl, which generated pBT2delfemB. All recombinant plasmids were introduced into *E. coli* DH5αby electroporation, and plasmids were introduced into *S. carnosus* via protoplast transformation [2]. Allelic replacement of the *femB* gene by *ermB* was performed as described elswhere (Brückner, [1]. For additional selective pressure, 4 ng/ml of lysostaphin (Genmedics GmbH) was added to the medium.

**Construction of *femB* expression plasmid**Primers FemBBglIIup-SD and FemBSmaIdo were used to amplify the *femB* gene from *S. carnosus* TM300 genomic DNA. The PCR product after digestion was purified and ligated to the BglII/SmaI sites of pPSHG5, a derivative of pPSHG3 [3] resulting in plasmid pPSHG5femB. Plasmids were introduced into *S. carnosus* via protoplast transformation.

**Antimicrobial susceptibility testing**

Antibiotic susceptibility was tested with agar disc diffusion assay or serial broth dilution method according to the guidelines of the National Committee for Clinical Laboratory Standards [4, 5]. Lysostaphin minimum inhibitory concentration assay was determined as described in an earlier study [6].

## **Purification and analysis of peptidoglycan (PGN)**

## Peptidoglycan (PGN) was isolated from *S. carnosus* TM300, *S. carnosus* Δ*femB*, and the complementary mutant as described in an earlier study [7]. Briefly, cells grown for 7 h and harvested by centrifugation were boiled with 5% SDS for 30 min, and broken with glass beads. Broken cells were washed SDS free and resuspended in 100 mM Tris-HCl (pH 7.2) containing 20 mM MgCl_2_ and treated with 10 µg/ml DNAse and 50 µg/ml RNAse for 2 h and subsequently with 100 µg/ml trypsin, 37 °C overnight. To remove wall teichoic acid, the PGN preparations were incubated with 48% hydrofluoric acid (HFA) for 24 h at 4°C while stirred after washing with water. PGN was harvested by centrifugation and washed several times with water until HFA was completely removed and lyophilized. Lyophilized PGN was resuspended in 25 mM sodium phosphate buffer (pH 6.8) to a final OD_578_ of 5.0, digested with mutanolysin overnight at 37 °C and analysed by HPLC as described earlier [7].

**Analysis of proteins in the supernatant, membrane and cytosolic fractions by SDS-PAGE**

Proteins were analyzed by tricine-12% sodium dodecyl sulfate polyacrylamide gel electrophoresis SDS-PAGE [8]. Based on OD_578nm_, adequate aliquots of the respective samples were centrifuged for 15 min at 4,000 x g at 4 °C and proteins of the culture supernatant were concentrated by using StrataClean™ Resin (Stratagene, La Jolla, CA, USA). The respective cell pellet was washed with PBS (7 mM Na_2_HPO_4_ × 2H_2_O, 3 mM NaH_2_PO_4_ × 2H_2_O, and 0.85% NaCl; pH 7.2), resuspended with loading dye (5-fold: 0.25 M Tris/HCl [pH 6.8] 50% [v/v] glycerol, 5% SDS, and 0.05% bromophenol blue) according to the cell wet weight (w/v) and heated for 5 min at 90 °C. After centrifugation at 17,500 x g for 3 min, equal amount of the supernatant was loaded on SDS gel. To isolate the cytosolic fractions, cell pellets were resuspended in buffer (20 mM Tris/HCl; pH 7.8) and brocken using glass beads (0.25–0.5 mm, Fast Prep, 4 times, Sigma–Aldrich). Brocken cells were centrifuged, and equal amount of the supernatant was loaded on the gel. Proteins were visualized by staining with Coomassie brilliant blue R-250.

**Western blot analysis**

Same amount of protein per lane was separated on 12% SDS-PAGE as described in an earlier study [8] and transferred to nitrocellulose for 1 h and 20 min at 350 mA and buffered in semi-dry transfer buffer using a Trans-Blot SD Semi-Dry Transfer Cell. The membrane was incubated for 2 h with 5% goat serum and diluted in tris-buffered saline (TBS) buffer containing 0.3% bovine serum albumin (BSA). For detection, membrane was incubated with the specific rabbit polyclonal anti-GAP-DH (1:2,500), anti-FbaA (1:2,500), anti-Eno (1:5,000), and anti-NDH-2 (1:10,000) for 1 h. Bound antibodies were detected by incubation with anti-rabbit alkaline phosphatase-labeled secondary antibody (1:20,000) in TBS buffer containing 0.3% BSA. For chemiluminescence detection, nitro blue tetrazolium/5-bromo-4-chloro-3-indolyl phosphate ready-to-use solution was used.

**Fluorescence microscopy**

##### Cells were grown in a BM medium without glucose but with 0.25 % galactose until the mid-exponential phase. Afterwards, 1 ml of cell suspension was harvested by centrifugation at 17,500 x g for 1 min and washed with 1 ml phosphate buffered saline (PBS) at 17,500 x g for 1 min. After washing, cells were resuspended in 250 μl PBS and incubated for 10 min at 37 °C with fluorescence-labeled vancomycin (BODIPY® FL, Invitrogen) for peptidoglycan labeling, FM 4-64 (Life Technologies) for plasma membrane staining, and DAPI (4’,6-diamidino-2-phenylindole, Life Technologies) for DNA staining. After incubation with the dyes for 10 min, cells were washed twice with PBS at 17,500 x g for 1 min. Fluorescence microscopy was performed with an upright microscope (Leica DM5500 B), and images were captured with a high-sensitivity camera (Leica DFC360 FX). Fluorescence quantification was performed using Leica Application Suite Advanced Fluorescence software and ImageJ software.

**RNA isolation and northern blot analysis**

Total RNA from *S. carnosus*, the isogenic *femB* mutant, and the complementary strain were isolated using the acid-phenol method described in earlier studies [9, 10] with some modifications [11]. Digoxigenin-labeled RNA probes were prepared by *in vitro* transcription with T7 RNA polymerase by using a PCR fragment as a template [10, 12], which was purified using the High Pure PCR Cleanup Micro Kit (Roche Diagnostics) and resolved from the column in 50 µl of double-distilled water. The PCR fragment was generated with the chromosomal DNA of *S. carnosus* TM300 and the respective oligonucleotides (Table 1). Northern blot experiments were performed as described in a previous study [11]. The digoxigenin-labeled RNA probe stored at -80 °C was used for hybridization, the signals of which were detected with an ECL-film (GE Healthcare) and scanned with a light scanner.

**Protein identification by mass spectrometry**

Protein spots were excised with the Proteome Works™ spot picker or manually by cutting a spot 2 mm in diameter and transferring it into 96-well microtiter plates. In-gel digestion with trypsin, extraction, and the spotting of the peptide solutions were done automatically using the Ettan Spot Handling Workstation. Peptide masses were measured by MALDI TOF spectrometry (MALDI-TOF Proteome Analyzer 4700, Applied Biosystems) as described in a previous study [13]. Peptide mass fingerprints were analyzed using GPS explorer with integrated MASCOT software (http://www.matrixscience.com) and the *S. carnosus* genome sequence. Criteria for an identification of a protein were a MASCOT score of greater than 49, a replicated identification result at the same position, and an observed molecular weight close to the predicted protein. Database searches were performed using GPS explorer software version 3.6 with organism-specific databases.

**Supporting Information Figures 1 and 2**

**Supporting Information Fig 1.** SDS-PAGE of culture supernatants of *S. carnosus* and various *femB* mutants. Strains were cultivated aerobically in BM for 4, 8 and 16h. after this time points the cell culture was adjusted to OD 4.0 and proteins from 3 ml culture supernatants were applied to the gel.

**Supporting Information Fig. 2.** 2D-PAGE of secretome of *S. carnosus* wt and its *femB* mutant. Because of the retarded growth of the mutant the samples were taken at different time points, 4 and 12 h for the wt and 8 and 16 h for the mutant. OD_578nm_ values as well as the protein quantity (μg/μl protein solution) are indicated.

**Supporting Information for Tables 1-4**

**Supporting Information Table 1**. Oligonucleotide primers used in this study

| **Primer** | **Sequence (5' to 3')*^a^*** |
| --- | --- |
| 1koFemBEcoRIup | CTACAGCT*GAATTC*GGAGCATTTACAGATCAAATGCC |
| 2koFemBSmaIdo | AAATTT*CCCGGG*TACCTTATGTTTATCTATCTTTG |
| 3koFemBSalIup | TTTAAA*GTCGAC*TGAAAGTAATCATACAACATTGC |
| 4koFemBEcoRVdo | TTTAAA*GATATC*GTTGAACACACGCAATAATCAATGTTAC |
| FemBBglIIup-SD | AAATTT*AGATCT*AAGGTAGAAGGGAAATAAATCAATG |
| FemBSmaIdo | TTTAAA*CCCGGG*TTTGATTCGCAGGTATTGCAATGTTG |
| Sca0039 *ahpC* forward | CACAGCAGACGCATATAACC |
| Sca0039 *ahpC* reverse | ctaatacgactcactatagggagacctactaagtcaagacctgg |
| Sca0404 forward | cgctaaattaagcgatgtcg |
| Sca0404 reverse | ctaatacgactcactatagggagagctgttgattgcatgatagc |
| Sca0424 *gapA* forward | GGTAGAATCGGTCGTTTAGC |
| Sca0424 *gapA* reverse | ctaatacgactcactatagggagatctgtgtggaccatcttgag |
| Sca0428 *eno* forward | CTGAAAGTGGTGCATTCGGT |
| Sca0428 *eno* reverse | ctaatacgactcactatagggagaagcaaatccaccttcgtcac |
| Sca0659 *bph* forward | gcagatcagacacaacaacc |
| Sca0659 *bph* reverse | ctaatacgactcactatagggagagtttgagcagcaggttgaac |
| Sca0983 *tkt* forward | CAGACACACTCAAGGTGTAG |
| Sca0983 *tkt* reverse | ctaatacgactcactatagggagatcttcacctaatggtgcacc |
| Sca1315 forward | CGTTGATGGATCACAAGAAG |
| Sca1315 reverse | ctaatacgactcactatagggagacagtagcaacttgtggttgg |
| Sca1598 *sceA* forward | gtagtatcatcagtagccac |
| Sca1598 *sceA* reverse | ctaatacgactcactatagggagaagcagttgcgtttgaaagtg |
| Sca1599 *sceD* forward | gtagcttcatcagcatcagcag |
| Sca1599 *sceD* reverse | ctaatacgactcactatagggagatcttgtactgcttcaggagc |
| Sca1790 *sceB* forward | gctacaactacaatcgctac |
| Sca1790 *sceB* reverse | ctaatacgactcactatagggagatggctagtttgcatgatagc |
| Sca2336 *katA* forward | CAGATGCTGAACGTGATATC |
| Sca2336 *katA* reverse | ctaatacgactcactatagggagaggattgtctttgtgattgcg |

*^a^* Restriction sites are marked in italics. The underlined sequences at the 5´-end represents the recognition site of the T7 RNA polymerase [12].

**Supporting Information Table 2**: MS-Data of identified spots of the *S. carnosus* TM300 extracellular proteome (Protein Score, Sequence Coverage %)

| Protein*^a^* | Function | *S. carnosus* TM300  gene ID | *S. aureus* N315 homolog  gene ID | Protein Score | Sequence  Coverage % |
| --- | --- | --- | --- | --- | --- |
| **Extracellular proteins** | |  |  |  |  |
| Bph | Bifunctional peptidoglycan hydrolase precursor | Sca_0659 | SA0905 *atlA* | 654 | 21 |
| SceA | SceA precursor | Sca_1598 | SA1898 *sceD*  SA2356 *isaA* | 177 | 13 |
| SceB | SceB precursor | Sca_1790 | SA2093 *ssaA* | 414 | 33 |
| SceD | SceD precursor | Sca_1599 | SA1898 *sceD* | 77 | 12 |
| Sca0404 | LysM familiy protein | Sca_0404 | - | 265 | 23 |
| Sca1919 | Hypothetical protein | Sca_1919 | SA0295 | 123 | 37 |
| **Cell wall anchored proteins** | |  |  |  |  |
| Sca2092 | Hypothetical protein | Sca_2092 | - | 179 | 28 |
| Sca2283 | Hypothetical protein | Sca_2283 | - | 65 | 50 |
| **Membrane proteins** | |  |  |  |  |
| AldA | Putative aldehyde dehydrogenase | Sca_2103 | SA0162 *aldA* | 469 | 11 |
| DnaK | Chaperone protein DnaK (Hsp70) homolog | Sca_1202 | SA1409 *dnaK* | 475 | 52 |
| EF-TU | Translational elongation factor TU homolog | Sca_0207 | SA0506 *tufA* | 406 | 52 |
| Eno | 2-phospho-D-glycerate hydrolase (enolase) homolog | Sca_0428 | SA0731 *eno* | 46 | 37 |
| Fhs | Formate-tetrahydrofolate ligase homolog | Sca_1337 | SA1553 *fhs* | 379 | 56 |
| GlpD | Aerobic glycerol-3-phosphate dehydrogenase homolog | Sca_0950 | SA1142 *glpD* | 296 | 46 |
| Mqo | Malate dehydrogenase (acceptor) homolog | Sca_1865 | SA2155 | 116 | 42 |
| Mqo2 | Putative malate:quinone oxidoreductase | Sca_2266 | SA2400 *mqo2* | 306 | 62 |
| Pfk | 6-phosphofructokinase homolog | Sca_1304 | SA1521 *pfk* | 21 | 10 |
| SdhB | Succinate dehydrogenase iron-sulfor protein subunit homolog | Sca_0767 | SA0996 *sdhB* | 158 | 33 |
| TpiA | Triosephosphate isomerase homolog | Sca_0426 | SA0729 *tpi* | 295 | 33 |
| Sca0366 | Polyglycerol phosphate synthase LtaS | Sca_0366 | SA0674 *ltaS* | 224 | 10 |
| Sca0979 | Hypothetical protein | Sca_0979 | SA1173 | 303 | 47 |
| Sca1371 | Hypothetical protein | Sca_1371 | SA1585 | 261 | 28 |
| Sca1543 | Repeat family protein | Sca_1543 | - | 352 | 10 |
| **Cytosolic proteins** | |  |  |  |  |
| AckA | Acetate kinase homolog | Sca_1316 | SA1533 | 456 | 46 |
| AhpC | Alkyl hydroperoxide reductase subunit C | Sca_0039 | SA0366 *ahpC* | 164 | 42 |
| AtpG | ATP synthase gamma chain homolog | Sca_1607 | SA1906 *atpG* | 583 | 54 |
| CapI | Capsular polysaccharide biosynthesis protein CapI | Sca_2349 | - | 397 | 44 |
| CitB | Aconitate hydratase (aconitase) homolog | Sca_0991 | SA1184 *citB* | 525 | 40 |
| CitZ | Citrate synthase II | Sca_1301 | SA1518 *citZ* | 145 | 31 |
| ClpL | Putative ATP-dependent Clp proteinase chain | Sca_1953 | SA2336 *clpL* | 377 | 57 |
| CysK | Cysteine synthase (O-acetylserine sulfhydrylase) homolog | Sca_0164 | SA0471 *cysK* | 404 | 53 |
| Drm | Putative phosphopentomutase | Sca_2011 | SA0134 *drm* | 152 | 17 |
| EF-G | Elongation factor EF-G | Sca_0206 | SA0505 *fus* | 473 | 44 |
| EF-TS | Putative elongation factor TS | Sca_0892 | SA1100 *tsf* | 153 | 59 |
| FabG | 3-oxoacyl-(acyl-carrier protein) reductase | Sca_0854 | SA1074 *fabG* | 511 | 71 |
| FabI | Putative trans-2-enoyl-ACP reductase | Sca_0612 | SA0869 *fabI* | 118 | 13 |
| GapA | Glyceraldehyde-3-phosphate dehydrogenase | Sca_0424 | SA0727 *gap* | 232 | 31 |
| GapB | Glyceraldehyde 3-phosphate dehydrogenase 2 | Sca_1293 | SA1510 *gapB* | 208 | 56 |
| GatB | Glutamyl-tRNAGln amidotransferase subunit B homolog | Sca_1471 |  |  |  |
| GroEL | GroEL protein homolog | Sca_1540 | SA1836 *groEL* | 297 | 22 |
| GrpE | Putative GrpE protein (HSP-70 cofactor) | Sca_1203 | SA1410 *grpE* | 217 | 28 |
| GuaB | Putative inositol-monophosphate dehydrogenase | Sca_0049 | SA0375 *guaB* | 251 | 42 |
| IleS | Putative isoleucyl-tRNA synthetase | Sca_0806 | SA1036 *ileS* | 218 | 30 |
| KatA | Catalase | Sca_2336 | SA1170 *katA* | 356 | 38 |
| LytM | Putative LytM-like peptidoglycan hydrolase | Sca_2377 | SA0265 *lytM* | 358 | 16 |
| PdhA | Pyruvate dehydrogenase E1 component alpha subunit homolog | Sca_0719 | SA0943 *pdhA* | 292 | 32 |
| PdhB | Pyruvate dehydrogenase E1 component beta subunit homolog | Sca_0720 | SA0944 *pdhB* | 400 | 50 |
| PdhC | Dihydrolipoamide acetyltransferase component of pyruvate dehydrogenase complex E2 | Sca_0721 | SA0945 *pdhC* | 166 | 33 |
| PdhD | Dihydrolipoamide dehydrogenase component of pyruvate dehydrogenase E3 | Sca_0722 | SA0946 *pdhD* | 549 | 51 |
| PlsX | Putative fatty acid/phospholipid synthesis protein PlsX | Sca_0852 | SA1072 *plsX* | 346 | 76 |
| PpdK | Pyruvate phosphate dikinase homolog | Sca_1694 | - | 554 | 41 |
| RocA | 1-pyrroline-5-carboxylate dehydrogenase homolog | Sca_0564 | SA2341 *rocA* | 229 | 6 |
| RplA | 50S ribosomal protein L1 homolog | Sca_0194 | SA0496 *rplA* | 174 | 10 |
| RplC | 50S ribosomal protein L3 homolog | Sca_1735 | SA2047 *rplC* | 153 | 45 |
| RplE | 50S ribosomal protein L5 homolog | Sca_1723 | SA2035 *rplE* | 145 | 10 |
| RplF | Probable 50S ribosomal protein L6 | Sca_1720 | SA2033 *rplF* | 144 | 10 |
| RplJ | 50S ribosomal protein L10 homolog | Sca_0195 | SA0497 *rplJ* | 374 | 88 |
| RplY | 50S ribosomal protein L25 | Sca_0151 | SA0459 *rplY* | 310 | 47 |
| RpoB | RNA polymerase beta chain homolog | Sca_0199 | SA0500 *rpoB* | 248 | 7 |
| RpsB | 30S ribosomal protein S2 | Sca_0891 | SA1099 *rpsB* | 311 | 10 |
| OdhA | Putative 2-oxoglutarate dehydrogenase E1 component | Sca_1058 | SA1245 *kgd* | 334 | 44 |
| SucD | Putative succinyl-CoA synthetase, alpha chain | Sca_0883 | SA1089 *sucD* | 369 | 37 |
| ThiI | Truncated putative thiamine biosynthesis ATP pyrophosphatase | Sca_1320 | SA1537 | 96 | 10 |
| Tig | Trigger factor homolog | Sca_1281 | SA1499 *tig* | 182 | 48 |
| Tkt | Putative transketolase | Sca_0983 | SA1177 *tkt* | 357 | 42 |
| UreC | UreC urease alpha subunit homolog | Sca_1782 | SA2084 *ureC* | 353 | 37 |
| Sca0081 | Putative intracellular protease/amidase | Sca_0081 | - | 259 | 49 |
| Sca0400 | Ribosome-associated protein | Sca_0400 | SA0707 | 164 | 47 |
| Sca0543 | Pyridine nucleotide-disulphide oxidoreductase | Sca_0543 | SA0799 | 356 | 55 |
| Sca0559 | Putative peptidyl-prolyl cis-trans isomerase | Sca_0559 | SA0815 | 168 | 21 |
| Sca0563 | NADH-dependent flavin oxidoreductase | Sca_0563 | SA0817 | 569 | 66 |
| Sca1315 | Hypothetical protein | Sca_1315 | SA1532 | 317 | 75 |
| Sca1318 | Thioredoxin peroxidase | Sca_1318 | SA1535 *tpx* | 278 | 67 |
| Sca1903 | Hypothetical protein | Sca_1903 | SA2479 | 226 | 6 |
| Sca1991 | Pyruvate oxidase | Sca_1991 | SA2327 | 444 | 48 |
| Sca2186 | Hypothetical protein | Sca_2186 | SA2164 | 415 | 61 |
| **Proteins with unknown localization** | |  |  |  |  |
| PykA | Pyruvate kinase homolog | Sca_1303 | SA1520 *pykA* | 22 | 10 |
| ThiD | Putative phosphomethylpyrimidine kinase | Sca_1595 | SA1896 *thiD* | 271 | 9 |
| Zwf | Putative glucose-6-phosphate 1-dehydrogenase | Sca_1130 | SA1336 | 259 | 30 |
| Sca0546 | NADH dehydrogenase | Sca_0546 | SA0802 | 462 | 61 |
| Sca2221 | Hypothetical protein | Sca_2221 | - | 294 | 44 |
| Sca2222 | Hypothetical protein | Sca_2222 | - | 151 | 25 |
| Sca2250 | Hypothetical protein | Sca_2250 | - | 61 | 24 |

**Supporting Information Table 3**: Identification of 82 protein spots in secretome of the *S. carnosus* TM300 and

predicted location

| **Protein*^a^*** | **Function** | ***S. carnosus* TM300**  **gene ID** | ***S. aureus* N315 homolog**  **gene ID** | **Signal-**  **peptide*^b^*** | **MW (kDa)^c^** | **pI*^d^*** |  |
| --- | --- | --- | --- | --- | --- | --- | --- |
| **Secreted proteins (with Signal Peptide)** | |  |  |  |  |  |  |
| AtlCS | Major autolysin precursor | Sca_0659 | SA0905 *atlA* | + | 133.1 | 9.2 |  |
| SceA | SceA precursor | Sca_1598 | SA1898 *sceD*  SA2356 *isaA* | + | 22.2 | 5.2 |  |
| SceB | SceB precursor | Sca_1790 | SA2093 *ssaA* | + | 25.3 | 7.9 |  |
| SceD | SceD precursor | Sca_1599 | SA1898 *sceD* | + | 22 | 3.9 |  |
| Sca0404 | LysM familiy protein | Sca_0404 | - | + | 32.4 | 6.1 |  |
| Sca0979 | Hypothetical protein | Sca_0979 | SA1173 | + | 39.3 | 7.3 |  |
| Sca1543 | Repeat family protein | Sca_1543 | - | + | 64.2 | 4.5 |  |
| Sca1919 | Hypothetical protein | Sca_1919 | SA0295 | + | 30.9 | 8.8 |  |
| Sca2221 | Hypothetical protein | Sca_2221 | - | + | 17.1 | 5.1 |  |
| Sca2222 | Hypothetical protein | Sca_2222 | - | + | 33.9 | 4.4 |  |
| Sca2250 | Hypothetical protein | Sca_2250 | - | + | 37.2 | 4.5 |  |
| **Cell wall anchored proteins (with SP + sorting sequence)** | |  |  |  |  |  |  |
| Sca2092 | Hypothetical protein | Sca_2092 | - | + | 48.1 | 7.9 |  |
| Sca2283 | Hypothetical protein | Sca_2283 | - | + | 90.2 | 3.4 |  |
| **Membrane localized (most likely)** | |  |  |  |  |  |  |
| Mqo | Malate dehydrogenase (acceptor) homolog | Sca_1865 | SA2155 | - | 54.8 | 5.6 |  |
| Mqo2 | Putative malate:quinone oxidoreductase | Sca_2266 | SA2400 m*qo2* | - | 55.5 | 5.4 |  |
| SdhB | Succinate dehydrogenase iron-sulfor protein subunit homolog | Sca_0767 | SA0996 *sdhB* | - | 30.8 | 6.5 |  |
| Sca0366 | Polyglycerol phosphate synthase LtaS | Sca_0366 | SA0674 *ltaS* | - | 74.3 | 9.0 |  |
| **Cytosolic proteins** | |  |  |  |  |  |  |
| AckA | Acetate kinase homolog | Sca_1316 | SA1533 | - | 43.6 | 5.5 |  |
| AhpC | Alkyl hydroperoxide reductase subunit C | Sca_0039 | SA0366 *ahpC* | - | 21.1 | 4.5 |  |
| AldA | Putative aldehyde dehydrogenase | Sca_2103 | SA0162 *aldA* | - | 55.4 | 4.9 |  |
| AtpG | ATP synthase gamma chain homolog | Sca_1607 | SA1906 *atpG* | - | 31.9 | 8.5 |  |
| CapI | Capsular polysaccharide biosynthesis protein CapI | Sca_2349 | - | - | 43.6 | 5.5 |  |
| CitB | Aconitate hydratase (aconitase) homolog | Sca_0991 | SA1184 *citB* | - | 98.8 | 4.7 |  |
| CitZ | Citrate synthase II | Sca_1301 | SA1518 *citZ* | - | 42.4 | 5.3 |  |
| ClpL | Putative ATP-dependent Clp proteinase chain | Sca_1953 | SA2336 *clpL* | - | 77.9 | 4.9 |  |
| CysK | Cysteine synthase (O-acetylserine sulfhydrylase) homolog | Sca_0164 | SA0471 *cysK* | - | 33.3 | 5.3 |  |
| DnaK | Chaperone protein DnaK (Hsp70) homolog | Sca_1202 | SA1409 *dnaK* | - | 65.9 | 4.5 |  |
| Drm | Putative phosphopentomutase | Sca_2011 | SA0134 *drm* | - | 44.4 | 4.7 |  |
| EF-G | Elongation factor EF-G | Sca_0206 | SA0505 *fus* | - | 76.8 | 4.7 |  |
| EF-TS | Putative elongation factor TS | Sca_0892 | SA1100 *tsf* | - | 32.6 | 5.1 |  |
| EF-TU | Translational elongation factor TU homolog | Sca_0207 | SA0506 *tufA* | - | 43.2 | 4.7 |  |
| Eno | | 2-phospho-D-glycerate hydrolase (enolase) homolog | Sca_0428 | SA0731 *eno* | - | 47.3 | 4.6 |
| FabG | 3-oxoacyl-(acyl-carrier protein) reductase | Sca_0854 | SA1074 *fabG* | - | 26.1 | 5.2 |  |
| FabI | Putative trans-2-enoyl-ACP reductase | Sca_0612 | SA0869 *fabI* | - | 28.0 | 5.5 |  |
| FbaA | fructose-bisphosphate aldolase | Sca_1628 | SA1927 *fbaA* | - | 31.0 | 4.9 |  |
| Fhs | Formate-tetrahydrofolate ligase homolog | Sca_1337 | SA1553 *fhs* | - | 59.9 | 5.4 |  |
| GapA | Glyceraldehyde-3-phosphate dehydrogenase | Sca_0424 | SA0727 *gap* | - | 36.3 | 4.7 |  |
| GapB | Glyceraldehyde 3-phosphate dehydrogenase 2 | Sca_1293 | SA1510 *gapB* | - | 36.9 | 5.2 |  |
| GatB | Glutamyl-tRNAGln amidotransferase subunit B homolog | Sca_1471 |  | - | 53.7 | 4.9 |  |
| GlpD | Aerobic glycerol-3-phosphate dehydrogenase homolog | Sca_0950 | SA1142 *glpD* | - | 62.7 | 5.8 |  |
| GroEL | GroEL protein homolog | Sca_1540 | SA1836 *groEL* | - | 57.8 | 8.6 |  |
| GrpE | Putative GrpE protein (HSP-70 cofactor) | Sca_1203 | SA1410 *grpE* | - | 23.0 | 4.3 |  |
| GuaB | Putative inositol-monophosphate dehydrogenase | Sca_0049 | SA0375 *guaB* | - | 52.8 | 5.5 |  |
| IleS | Putative isoleucyl-tRNA synthetase | Sca_0806 | SA1036 *ileS* | - | 105.3 | 5.1 |  |
| KatA | Catalase | Sca_2336 | SA1170 *katA* | - | 57.2 | 5.3 |  |
| LytM | Putative LytM-like peptidoglycan hydrolase | Sca_2377 | SA0265 *lytM* | - | 35.6 | 4.3 |  |
| Ndh-2 | NADH dehydrogenase | Sca_0546 | SA0802 ndh2 | - | 44.0 | 5.6 |  |
| PdhB | Pyruvate dehydrogenase E1 component beta subunit homolog | Sca_0720 | SA0944 *pdhB* | - | 35.2 | 4.6 |  |
| PdhC | Dihydrolipoamide acetyltransferase component of pyruvate dehydrogenase complex E2 | Sca_0721 | SA0945 *pdhC* | - | 48.1 | 4.7 |  |
| PdhD | Dihydrolipoamide dehydrogenase component of pyruvate dehydrogenase E3 | Sca_0722 | SA0946 *pdhD* | - | 49.7 | 5.0 |  |
| PlsX | Putative fatty acid/phospholipid synthesis protein PlsX | Sca_0852 | SA1072 *plsX* | - | 35.1 | 6.3 |  |
| PpdK | Pyruvate phosphate dikinase homolog | Sca_1694 | - | - | 94.5 | 4.7 |  |
| PykA | Pyruvate kinase homolog | Sca_1303 | SA1520 *pykA* | - | 63.2 | 5.1 |  |
| RocA | 1-pyrroline-5-carboxylate dehydrogenase homolog | Sca_0564 | SA2341 *rocA* | - | 56.9 | 5.1 |  |
| RplA | 50S ribosomal protein L1 homolog | Sca_0194 | SA0496 *rplA* | - | 24.8 | 8.8 |  |
| RplC | 50S ribosomal protein L3 homolog | Sca_1735 | SA2047 *rplC* | - | 23.7 | 9.6 |  |
| RplE | 50S ribosomal protein L5 homolog | Sca_1723 | SA2035 *rplE* | - | 20.2 | 9.0 |  |
| RplF | Probable 50S ribosomal protein L6 | Sca_1720 | SA2033 *rplF* | - | 19.6 | 9.5 |  |
| RplJ | 50S ribosomal protein L10 homolog | Sca_0195 | SA0497 *rplJ* | - | 17.8 | 5.1 |  |
| RplY | 50S ribosomal protein L25 | Sca_0151 | SA0459 *rplY* | - | 23.2 | 4.3 |  |
| RpoB | RNA polymerase beta chain homolog | Sca_0199 | SA0500 *rpoB* | - | 133.2 | 4.9 |  |
| RpsB | 30S ribosomal protein S2 | Sca_0891 | SA1099 *rpsB* | - | 29.6 | 5.5 |  |
| OdhA | Putative 2-oxoglutarate dehydrogenase E1 component | Sca_1058 | SA1245 *kgd* | - | 105.6 | 5.3 |  |
| SucD | Putative succinyl-CoA synthetase, alpha chain | Sca_0883 | SA1089 *sucD* | - | 31.9 | 5.0 |  |
| ThiI | Truncated putative thiamine biosynthesis ATP pyrophosphatase | Sca_1320 | SA1537 | - | 27.2 | 5.6 |  |
| ThiD | Putative phosphomethylpyrimidine kinase | Sca_1595 | SA1896 *thiD* | - | 29.8 | 5.3 |  |
| Tig | Trigger factor homolog | Sca_1281 | SA1499 *tig* | - | 49.5 | 4.3 |  |
| Tkt | Putative transketolase | Sca_0983 | SA1177 *tkt* | - | 72.8 | 5.0 |  |
| TpiA | Triosephosphate isomerase homolog | Sca_0426 | SA0729 *tpi* | - | 27.5 | 4.8 |  |
| UreC | UreC urease alpha subunit homolog | Sca_1782 | SA2084 *ureC* | - | 62.4 | 5.3 |  |
| Zwf | Putative glucose-6-phosphate 1-dehydrogenase | Sca_1130 | SA1336 | - | 56.9 | 5.8 |  |
| Sca0081 | Putative intracellular protease/amidase | Sca_0081 | - | - | 25.7 | 6.7 |  |
| Sca0400 | Ribosome-associated protein | Sca_0400 | SA0707 | - | 21.9 | 5.5 |  |
| Sca0543 | Pyridine nucleotide-disulphide oxidoreductase | Sca_0543 | SA0799 | - | 39.7 | 5.7 |  |
| Sca0559 | Putative peptidyl-prolyl cis-trans isomerase | Sca_0559 | SA0815 | - | 21.8 | 4.4 |  |
| Sca0563 | NADH-dependent flavin oxidoreductase | Sca_0563 | SA0817 | - | 42.3 | 5.3 |  |
| Sca1315 | Hypothetical protein | Sca_1315 | SA1532 | - | 18.83 | 5.2 |  |
| Sca1318 | Thioredoxin peroxidase | Sca_1318 | SA1535 *tpx* | - | 18.2 | 4.4 |  |
| Sca1371 | Proline dehydrogenase homolog | Sca_1371 | SA1585 | - | 37.3 | 5.5 |  |
| Sca1903 | Hypothetical protein | Sca_1903 | SA2479 | - | 19.1 | 4.6 |  |
| Sca1991 | Pyruvate oxidase | Sca_1991 | SA2327 | - | 63.6 | 6.1 |  |
| Sca2186 | Hypothetical protein | Sca_2186 | SA2164 | - | 36.4 | 5.7 |  |

***^-----------------------------------------------------------------------------------------------------------------------------------------------------------------------------------------------------------------------------------------------------------------^***

***^a^*** Subcellular localization was predicted using PSORTb. ***^b^*** Typical signal sequence was predicted using SignalP. ***^c^*** Theoretical

molecular weight (MW) and ***^d^*** pI for mature proteins without signal sequence were calculated using MW/pI tools.

**Supporting Information Table 4.** Northern analysis of selected genes encoding cytoplasmic and secreted proteins

| **Gene** | **Gene accession no. *S. carnosus* TM300** | **Protein name** | ***S. aureus* N315 ortholog** | **WT** | **Δ*femB*** | **Δ*femB* (comp)** |
| --- | --- | --- | --- | --- | --- | --- |
| **Cytoplasmic proteins Transcription level** | | | | | | |
| *ahpC* | Sca0039 | alkyl hydroperoxide reductase subunit C | SA0366 (*ahpC*) | **1x** | **1x** | **1x** |
| *eno* | Sca0428 | enolase | SA0731 (*eno*) | **1x** | **1x** | **1x** |
| *gapA* | Sca0424 | glyceraldehyde-3-phosphate dehydrogenase | SA0727 (*gap*) | **1x** | **1x** | **1x** |
| *katA* | Sca2336 | catalase | SA1170 (*katA*) | **1x** | **1x** | **1x** |
| *tkt* | Sca0983 | putative transketolase | SA1177 (*tkt*) | **1x** | **1x** | **1x** |
| *sca1315* | Sca1315 | hypothetical protein | SA1532 (*h.p.*) | **1x** | **1x** | **1x** |
| **Secreted proteins Transcription level** | | | | | | |
| *sceA* | Sca1598 | SceA protein | SA1898 (*h.p.*) | **1x** | **2x ⇧** | **1x** |
| *sceB* | Sca1790 | SceB protein | SA2093 (*ssaA*) | **1x** | **2x ⇧** | **1x** |
| *sceD* | Sca1599 | SceD protein | SA1898 (*h.p.*) | **1x** | **2x ⇧** | **1x** |
| *atlCS* | Sca0659 | major autolysin (bifunctional PGN hydrolase) | SA0905 (*atlA*) | **1x** | **5x ⇧** | **1x** |
| *sca0404* | Sca0404 | putative LysM family protein | SA2093 (*ssaA*) | **1x** | **5x ⇧** | **1x** |

Data are based on published sequence of *S. carnosus* TM300 [14] and *S. aureus* N315 [15].

**1x**: transcription level is 100%; **2 or 5x ⇧** transcription level is 2 - 5 fold upregulated compared to **1x**.

**References**

[1] Brückner, R., Gene replacement in *Staphylococcus carnosus* and *Staphylococcus xylosus*. *FEMS Microbiol Lett* 1997, *151*, 1-8.

[2] Götz, F., Schumacher, B., Improvements of protoplast transformation in *Staphylococcus carnosus*. *FEMS Microbiol Lett* 1987, *40*, 285-288.

[3] Krismer, B. A., *Mikrobielle Genetik*, Eberhard-Karls-Universität, Thesis, 190, Tübingen 1999.

[4] NCCLS, Performance Standards for Antimicrobial Disk Susceptibility Tests; Approved Standard M2-A7, 7th ed. *National Committee for Clinical Laboratory Standards, Wayne, Pa* 2002.

[5] NCCLS, Methods for Dilution Antimicrobial Susceptibility Tests for Bacteria That Grow Aerobically; Approved Standard M7-A5, 5th ed. *National Committee for Clinical Laboratory Standards, Wayne, Pa* 2002.

[6] Kusuma, C. M., Kokai-Kun, J. F., Comparison of four methods for determining lysostaphin susceptibility of various strains of *Staphylococcus aureus*. *Antimicrob Agents Chemother* 2005, *49*, 3256-3263.

[7] de Jonge, B. L., Chang, Y. S., Gage, D., Tomasz, A., Peptidoglycan composition in heterogeneous Tn551 mutants of a methicillin-resistant *Staphylococcus aureus* strain. *Journal of Biological Chemistry* 1992, *267*, 11255-11259.

[8] Schägger, H., von Jagow, G., Tricine-sodium dodecyl sulfate-polyacrylamide gel electrophoresis for the sepatation of proteins in the range from 1 to 100 kDa. *Anal Biochem* 1987, *166*, 368-379.

[9] Majumdar, D., Avissar, Y. J., Wyche, J. H., Simultaneous and rapid isolation of bacterial and eukaryotic DNA and RNA: a new approach for isolating DNA. *Biotechniques* 1991, *11*, 94-101.

[10] Gertz, S., Engelmann, S., Schmid, R., Ohlsen, K.*, et al.*, Regulation of sigmaB-dependent transcription of *sigB* and *asp23* in two different *Staphylococcus aureus* strains. *Mol Gen Genet* 1999, *261*, 558-566.

[11] Fuchs, S., Pane-Farre, J., Kohler, C., Hecker, M., Engelmann, S., Anaerobic gene expression in *Staphylococcus aureus*. *J Bacteriol* 2007, *189*, 4275-4289.

[12] Jorgensen, E. D., Durbin, R. K., Risman, S. S., McAllister, W. T., Specific contacts between the bacteriophage T3, T7, and SP6 RNA polymerases and their promoters. *J Biol Chem* 1991, *266*, 645-651.

[13] Eymann, C., Dreisbach, A., Albrecht, D., Bernhardt, J.*, et al.*, A comprehensive proteome map of growing *Bacillus subtilis* cells. *Proteomics* 2004, *4*, 2849-2876.

[14] Rosenstein, R., Nerz, C., Biswas, L., Resch, A.*, et al.*, Genome analysis of the meat starter culture bacterium *Staphylococcus carnosus* TM300. *Applied and environmental microbiology* 2009, *75*, 811-822.

[15] Kuroda, M., Ohta, T., Uchiyama, I., Baba, T.*, et al.*, Whole genome sequencing of meticillin-resistant *Staphylococcus aureus*. *Lancet* 2001, *357*, 1225-1240.
